# Severe Chronic Kidney Disease Presenting as Asymptomatic Normocytic Anemia in a Child

**DOI:** 10.7759/cureus.108248

**Published:** 2026-05-04

**Authors:** Vaishali Benaka Hebbar, Pratibha Patil, Amrut Savadkar

**Affiliations:** 1 Pediatrics, Community Medical Center, Tracy, USA; 2 Internal Medicine, Dignity Health Saint Joseph Medical Center, Stockton, USA

**Keywords:** acute kidney injury, anemia, causes of anemia, chronic kidney disease (ckd), pediatrics, pediatrics patient, screening

## Abstract

Anemia is a common finding in pediatric practice and is most often attributed to nutritional deficiency; however, it may also represent the earliest manifestation of underlying systemic disease. We describe the case of a six-year-old girl who presented for a routine well-child visit and was found to have severe normocytic, normochromic anemia despite appearing clinically asymptomatic. Subsequent laboratory evaluation revealed profound renal dysfunction with metabolic acidosis and electrolyte abnormalities. Renal imaging demonstrated bilaterally small kidneys without hydronephrosis, consistent with advanced chronic kidney disease (CKD) likely due to congenital renal hypoplasia. The patient required emergent initiation of hemodialysis and was later transitioned to long-term renal replacement therapy. This case underscores the importance of maintaining a broad differential diagnosis when evaluating unexplained anemia in children and highlights that CKD may remain clinically silent until late stages, with anemia serving as a critical early diagnostic clue.

## Introduction

Anemia is among the most frequently encountered hematologic abnormalities in pediatric practice and encompasses a broad differential diagnosis. Iron deficiency remains the leading cause worldwide, particularly among young children with inadequate dietary intake or increased nutritional requirements [1,2]. However, anemia may also represent an early manifestation of underlying chronic systemic disorders, including chronic kidney disease (CKD), inflammatory conditions, hemolytic diseases, and bone marrow pathology [3,4]. Although nutritional deficiency accounts for the majority of pediatric anemia cases, non-nutritional etiologies should be carefully considered in children presenting with severe or normocytic anemia, especially when associated with concerning systemic findings [3,4]. The global prevalence of anemia among children aged 6-59 months is estimated at approximately 42.6%, with a disproportionately higher burden in low-resource settings [5].

Anemia in CKD is primarily due to inadequate production of erythropoietin, a hormone essential for red blood cell production. As renal function declines, erythropoietin deficiency becomes more pronounced, resulting in progressive anemia. In pediatric populations, the prevalence of anemia increases with CKD severity and is particularly common in advanced stages. [6,7]

CKD in children is most commonly caused by congenital anomalies of the kidney and urinary tract (CAKUT), including renal hypoplasia and dysplasia [8,9. These structural abnormalities may remain clinically silent for extended periods because compensatory renal function in early life masks the decline in kidney function.

We report a case of a previously asymptomatic child who was found to have severe normocytic anemia during routine evaluation and was ultimately diagnosed with advanced CKD. This case highlights the importance of considering systemic disease in children presenting with unexplained anemia and underscores the role of careful clinical evaluation in identifying underlying chronic kidney pathology.

## Case presentation

A six-year-old girl presented to the clinic for a routine well-child visit and catch-up immunizations. She had no complaints and denied fatigue, pallor, decreased appetite, or reduced activity. On examination, her height was 1.03 m (<3rd percentile), weight was 34 lb (<3rd percentile), BMI was at the 39th percentile, heart rate was 105 beats/min, respiratory rate was 23 breaths/min, blood pressure was 116/82 mmHg (>95th percentile), and oxygen saturation was 99%.

Physical examination revealed mild pallor but was otherwise unremarkable. There was no hepatosplenomegaly, edema, or lymphadenopathy. Family history was negative for anemia and known kidney disease. Routine point-of-care hemoglobin screening showed a hemoglobin level of 6.4 g/dL. A confirmatory complete blood count demonstrated normocytic, normochromic anemia. 

Laboratory studies revealed a ferritin level of 37 ng/mL, transferrin saturation of 16%, and total iron-binding capacity (TIBC) of 256 µg/dL. These findings were suggestive of anemia of chronic disease rather than iron deficiency. Red blood cell indices showed a mean corpuscular volume (MCV) of 84.4 fL, a mean corpuscular hemoglobin concentration (MCHC) of 30.7 g/dL, and a red cell distribution width (RDW) of 14.7%.

Given the severity of anemia, the patient was urgently referred to pediatric hematology. Repeat laboratory evaluation in the specialist's office demonstrated markedly elevated blood urea nitrogen and serum creatinine levels, along with significant electrolyte abnormalities (Table 1). The estimated glomerular filtration rate (eGFR) was approximately 6.3 mL/min/1.73 m², consistent with severe renal dysfunction.

**Table 1:** Initial laboratory tests HB, hemoglobin; HCT: hematocrit; APTT, activated partial thromboplastin time; INR, international normalized ratio; WBC, white blood cell count; BUN, blood urea nitrogen; CO₂, bicarbonate/total carbon dioxide

Test	Result	Reference range
WBC	3.6	4.5-13.5 K/µL
HB	6.4	11.5-15.5 g/dL
HCT	20.4%	35.0%-45.0%
Sodium	138	135-145 mmol/L
Potassium	4.4	3.5-5.0 mmol/L
CO₂	14	22-28mmol/L
Anion gap	23	8-16 mmol/L
BUN	106	5-18 mg/dL
Creatinine	6.71	0.3-1.10 mg/dL
Calcium	6	8.8-10.8 mg/dL
Magnesium	2.5	1.7-2.43 mg/dL
Phosphorus	9.6	4.0-6.0 mg/dL
APTT	29.7	25-35 sec
Prothrombin time	15.4	11-14 sec
INR	1.2	0.8-1.2

The patient was directed to the emergency department for urgent evaluation and was subsequently admitted. Laboratory results obtained during hospitalization are presented in Table 2.

**Table 2:** Laboratory results obtained during hospitalization WBC, white blood cell count; ANC, absolute neutrophil count; ALC, absolute lymphocyte count; HGB, hemoglobin; HCT, hematocrit; PLT, platelet count; Na, sodium; K, potassium; Cl, chloride; CO₂, bicarbonate/total carbon dioxide; BUN, blood urea nitrogen; CREAT, serum creatinine; Ca, calcium; Mg, magnesium; PO₄, phosphorus (phosphate); DOA, day of admission

Lab test	Day 7	Day 6	Day 5	Day 4	Day 3	Day 2	Day 1	DOA
WBC (4.5-13.5) K/µL	3.3	2.4	2.0	2.4	2.2	2.3	2.3	3.7
ANC (1.50-8.50)×10⁹/L	1.21	0.86	0.96		-	1.15	1.33	2.98
ALC (1.50-8.50)×10⁹/L	1.64	1.22	0.74	-	0.71	0.71	0.58	0.41
HGB (11.5-15.5g/dL)	8.4	8.7	8.5	8.9	8.6	8.8	8.1	8.3
HCT (35%-45%)	26.8	27.5	26.3	27.5	26.6	26.6	24.3	25
PLT (140-450)×10⁹/L	143	119	95	101	125	119	142	179
NA (135-145mmol/L)	142	147	139	140	-	141	141	149
K (3.5-5.0mmol/L)	3.9	3.4	3.9	4.6	3.9	3.9	3.8	3.8
Cl (101-110mmol/L)	109	102	107	108	109	109	105	112
CO_2_ (22-28mmol/L)	18	21	12	14	14	14	15	13
BUN (8-16mg/dL)	69	48	80	50	53	53	78	108
CREAT (0.3-1.10mg/dL)	4.05	3.03	4.29	3.4	3.83	3.83	4.92	6.15
CA (8.8-10.8mg/dL)	7.9	7.3	7.6	7.2	7.7	7.7	7.9	7.4
MG (1.7-2.1mg/dL)	2.4	2.3	2.6	2.1	2.1	2.2	2.2	2.3
PO_4_ (4.1-5.9mg/dL)	4.6	-	5.3	5.3	5.9	4.7	5.9	7.4

Renal ultrasound demonstrated bilaterally small kidneys without hydronephrosis, consistent with CKD. Additional laboratory investigations were performed to evaluate possible causes of renal failure. Autoimmune and immunologic testing, including antinuclear antibody (ANA), anti-double-stranded DNA antibodies, proteinase 3-antineutrophil cytoplasmic antibody (PR3-ANCA), myeloperoxidase-antineutrophil cytoplasmic antibody (MPO-ANCA), complement C3, complement C4, and anti-glomerular basement membrane antibodies, were all negative. Urinalysis revealed significant proteinuria with a protein-to-creatinine ratio of 3664 mg/g, while serum albumin remained within normal limits.

These findings suggested that advanced CKD was likely related to bilateral renal hypoplasia. Although congenital renal hypoplasia was considered the most likely etiology, other conditions associated with small kidneys, including nephronophthisis, remain part of the differential diagnosis. Genetic testing is currently pending for further evaluation.

The patient received a packed red blood cell transfusion, erythropoietin therapy (50 units/kg), and intravenous calcium supplementation for electrolyte abnormalities. Due to the severity of renal dysfunction, hemodialysis was initiated with one-hour sessions daily and gradually escalated to two hours and then three hours per session. As the patient’s electrolyte abnormalities improved, the frequency of dialysis was reduced to alternate days. During hospitalization, the patient remained clinically stable.

She was discharged home with plans for hemodialysis three times weekly and was later transitioned to peritoneal dialysis for long-term management. She was also started on vitamin D therapy for secondary hyperparathyroidism associated with CKD-related metabolic bone disease.

## Discussion

Anemia is a common finding in pediatric practice, and iron deficiency remains the most frequent etiology. However, normocytic, normochromic anemia should prompt consideration of alternative systemic causes, including CKD. In this case, the severity of anemia combined with normal iron indices suggested anemia of chronic disease rather than nutritional deficiency, prompting further evaluation.

Anemia in CKD primarily results from inadequate erythropoietin production due to progressive nephron loss. Additional contributors include chronic inflammation, impaired iron utilization, reduced erythrocyte lifespan, and metabolic derangements. In children, the degree of anemia generally correlates with declining renal function and is most pronounced in advanced CKD. [6,7]

CAKUT represents the most common cause of CKD in children. Among these, renal hypoplasia and renal dysplasia are important structural abnormalities that may lead to progressive nephron loss and eventual renal insufficiency [8,9]. These conditions may remain clinically silent for several years, particularly in cases where residual renal function is sufficient to maintain metabolic homeostasis during early childhood. As a result, many children are diagnosed only after complications such as growth failure, electrolyte abnormalities, or anemia become evident.

This patient’s presentation illustrates how CKD may remain clinically silent until significant organ dysfunction is present. Despite severe renal impairment, the child had minimal symptoms and was identified only after routine screening revealed marked anemia. Findings supporting chronicity included bilaterally small kidneys on ultrasonography, persistent growth failure, and longstanding metabolic abnormalities, favoring CKD over acute kidney injury.

Although bilateral renal hypoplasia was considered the most likely underlying etiology, the differential diagnosis for small kidneys associated with CKD in children remains broad. Conditions such as nephronophthisis, chronic glomerulopathies, and hereditary renal disorders must also be considered. Nephronophthisis is a relatively common genetic cause of end-stage renal disease in children and may present during school age with nonspecific findings, including anemia, polyuria, growth retardation, or incidentally detected renal dysfunction. In this patient, the absence of significant extrarenal manifestations and imaging findings consistent with small kidneys supported the possibility of congenital renal hypoplasia; however, definitive differentiation requires genetic evaluation or histopathologic confirmation. Renal biopsy was not pursued in this case because of the small size of the kidneys and the high likelihood of extensive fibrosis, which would limit diagnostic yield [10-12].

Growth impairment provided additional evidence of long-standing disease. Linear growth failure in CKD is multifactorial and often reflects chronic metabolic acidosis, disturbances in calcium-phosphorus metabolism, hormonal resistance, and inadequate nutrition. In this patient, persistent height and weight measurements below the third percentile further supported a chronic disease process.

This case underscores the diagnostic value of systematically evaluating unexplained normocytic anemia in children. While nutritional causes are common, anemia may be the earliest detectable manifestation of serious systemic disease, including CKD. Recognition of this presentation in primary care settings is critical, as early referral and intervention can facilitate timely management of renal failure and associated complications.

Several limitations should be acknowledged in this case. First, the definitive underlying cause of the patient’s renal disease was not confirmed with advanced imaging or histologic evaluation, such as renal biopsy or DMSA scanning. Renal biopsy was deferred due to the small size of the kidneys and the likelihood of extensive fibrosis from long-standing disease.

Second, a comprehensive hematologic evaluation was limited because of the urgency of the patient’s clinical condition. Additional studies, such as reticulocyte count, peripheral smear, vitamin B12 levels, and lactate dehydrogenase, were not obtained prior to initiating treatment. Long-term follow-up data, including transplant evaluation, were not available.

Despite these limitations, the available clinical and laboratory findings strongly supported advanced CKD as the underlying cause of the patient’s anemia.

## Conclusions

This case illustrates how severe CKD in children may remain clinically silent until advanced stages and may first present as unexplained anemia. Although iron deficiency is the most common cause of anemia in children, normocytic anemia should prompt further evaluation of systemic conditions, including CKD.

Early recognition and timely referral are critical for initiating appropriate management and preventing complications such as growth failure, metabolic bone disease, and cardiovascular morbidity. Clinicians should maintain a broad differential diagnosis when evaluating anemia, particularly when initial findings do not clearly indicate nutritional deficiency.
